# Early Electrophysiological Disintegration of Hippocampal Neural Networks in a Novel Locus Coeruleus Tau-Seeding Mouse Model of Alzheimer's Disease

**DOI:** 10.1155/2019/6981268

**Published:** 2019-06-12

**Authors:** A. Ahnaou, C. Walsh, N. V. Manyakov, S. A. Youssef, W. H. Drinkenburg

**Affiliations:** Department of Neuroscience Discovery, Janssen Research & Development, Janssen Pharmaceutica NV, Turnhoutseweg 30, B-2340 Beerse, Belgium

## Abstract

Alzheimer's disease (AD) is a progressive, neurodegenerative disease characterized by loss of synapses and disrupted functional connectivity (FC) across different brain regions. Early in AD progression, tau pathology is found in the locus coeruleus (LC) prior to amyloid-induced exacerbation of clinical symptoms. Here, a tau-seeding model in which preformed synthetic tau fibrils (K18) were unilaterally injected into the LC of P301L mice, equipped with multichannel electrodes for recording EEG in frontal cortical and CA1-CA3 hippocampal areas, was used to longitudinally quantify over 20 weeks of functional network dynamics in (1) power spectra; (2) FC using intra- and intersite phase-amplitude theta-gamma coupling (PAC); (3) coherence, partial coherence, and global coherent network efficiency (Eglob) estimates; and (4) the directionality of functional connectivity using extended partial direct coherence (PDC). A sustained leftward shift in the theta peak frequency was found early in the power spectra of hippocampal CA1 networks ipsilateral to the injection site. Strikingly, hippocampal CA1 coherence and Eglob measures were impaired in K18-treated animals. Estimation of instantaneous EEG amplitudes revealed deficiency in the propagation directionality of gamma oscillations in the CA1 circuit. Impaired PAC strength evidenced by decreased modulation of the theta frequency phase on gamma frequency amplitude further confirms impairments of the neural CA1 network. The present results demonstrate early dysfunctional hippocampal networks, despite no spreading tau pathology to the hippocampus and frontal cortex. The ability of the K18 seed in the brainstem LC to elicit such robust functional alterations in distant hippocampal structures in the absence of pathology challenges the classic view that tau pathology spread to an area is necessary to elicit functional impairments in that area.

## 1. Introduction

Alzheimer's disease (AD) is the most common cause of dementia, characterized by a progressive loss of cognitive function and, eventually, in the late stages of the disease, loss of basic motor functions such as swallowing [1, 2]. Currently, the only treatments available for AD are aimed at reducing the severity of cognitive impairments; there are no available treatments which halt, or even slow, the progression of the disease. This may soon result in an unmanageable public health disaster with an even heavier socioeconomic burden [3]. The main pathological hallmarks of AD are the formation of two types of lesions in the brain, extracellular plaques of amyloid beta (A*β*), and intracellular neurofibrillary tangles (NFT) of tau protein, leading to widespread neurodegeneration and atrophy of the brain [4, 5]. Until relatively recently, pharma drug discovery in the field of AD has been focused on therapies that reduce the levels of soluble or insoluble A*β* in the brain; however, numerous high-profile late-stage failures in clinical trials have resulted in a shift in focus to reducing the levels of tau in the brain far earlier in the presymptomatic stage of the disease [6]. A great deal of work is currently going on to investigate biomarkers of deteriorating brain function that could aid early diagnosis and act as indicators of therapeutic efficacy [7–11]. The back translational value of using functional biomarkers in preclinical AD drug discovery could be extremely high; the ability to predict whether an experimental therapeutic compound will have a functional benefit in humans before costly clinical testing would save the pharmaceutical industry time and money and allow discovery scientists to better identify the best compounds to take to the next stage [11].

Another obstacle that AD drug discovery must overcome is the lack of suitable AD animal models available for preclinical research. Recent high-profile failures of late-stage AD compounds have led many in the field to critically analyze the translatability of currently available AD mouse models and focus on earlier disease interventions, prior to the onset of symptoms. To date, there have been no mutations found in the tau gene that cause spontaneous AD; however, several proaggregation tau mutations have been discovered for another tauopathy, frontotemporal dementia with parkinsonism linked to chromosome 17 (FTDP-17), and many of these have been used to create tau transgenic mice [12]. Two of the most common tau mutations used in Alzheimer's mouse models are the P301L and P301S mutations. Both P301L and P301S mutations affect only 4 repeat tau isoforms as they occur within exon 10 of the tau sequence [13, 14]. Depending on the gene promoter under which these transgenes are expressed, tau pathology in the brains of these animals can develop primarily in the hindbrain or the forebrain. JNPL3 and rTg4510 mice both express the P301L mutation, but while JNPL3 mice express the transgene under a mouse prion promoter and have primarily hindbrain pathology [15], rTg4510 mice express it under a CaMK-II promoter, resulting in a predominantly forebrain expression [16]. It is for this reason that severe motor impairments are present in JNPL3 mice, yet absent in rTg4510 mice [15, 17]. The spatiotemporal progression of tau pathology in Alzheimer's disease was shown by [18] to vary very little between individuals, and unlike amyloid pathology which develops diffusely throughout the neocortex [19], tau pathology has been shown to develop in specific, anatomically connected regions of the brain [20]. This exact neuropathological staging has not been replicated in any mouse models, and without understanding the exact cause of the development of tau pathology in these areas, it will not be possible to perfectly model AD in mice. It has also recently been hypothesized that in AD, pathological tau proteins spread from affected regions like infectious proteins called prions and it is this spread of tau pathology to an area of the brain that results in the functional changes seen in this newly affected area [21].

An alternative novel disease model of AD in animals involves a seeding approach, which templates and spreads the pathology following the injection of aggregates of A*β* or tau protein into an area of the animal's brain [22]. In the case of tau, this pathology has been shown to spread to anatomically connected areas, similar to the pathological spread of tau in AD [23], and seeding tau aggregation in the CA1 hippocampal area impaired neuronal network dynamics in the seeded area [24]. It was recently discovered that the tau pathology in AD may in fact begin far earlier than expected, during adolescence, in the brainstem locus coeruleus (LC), before any clinical symptoms or concomitant cerebral amyloid pathology was evident [25, 26]. This led to the novel hypothesis that the pathologic process of AD is initiated by tau pathology in the LC, which is then transported via anatomically connected neurons to the medial temporal lobe to trigger subsequent neuropathologic changes associated with amyloid deposition before any clinical symptoms, or concomitant cerebral amyloid pathology was observed [27]. The LC complex is a group of small nuclei located deep in the pons and is the sole source of noradrenaline to most brain regions. Noradrenaline facilitates the interactions between networks, and the LC plays an important role in cognitive functions, including memory consolidation and retrieval [28]. Structural imaging studies have investigated changes within the LC with aging and AD. Neuronal loss and atrophy of the LC occur during aging and are early events in AD and correlate with cognitive performance [29]. Subsequently, K18 tau aggregates have been unilaterally injected into the LC of PS19 tau transgenic mice [30], which present with the P301S mutation [31], effectively seeding pathology, which spreads to anatomically connected areas similarly to what is seen in AD, and resulted in cell loss in the injected LC [30]. This seeding model seems to currently be the most effective animal model for the earliest stages of AD, as tau pathology initiates solely in the locus coeruleus, instead of diffusely within the hindbrain as is seen in the JNPL3 mouse [15]. One caveat to this is that surprisingly, there was no tau pathology spread to either the entorhinal cortex or the hippocampus, two regions affected early in AD [18]. The authors could not explain this discrepancy but suggest the possibility that development of tau pathology in these regions may be independent of tau pathology developing in the brainstem [30].

Both A*β* and tau protein have been shown to cause electrophysiological alterations in the brain prior to neurodegeneration [32, 33], and unlike many modern in vivo animal brain imaging techniques, pharmaco-EEG techniques are widely available and well validated for use in rodents [34]. Several hallmark EEG alterations have been demonstrated in AD and in some AD mouse models including the following: a shift from high-frequency oscillations to low-frequency oscillations, resulting in a “slowing,” altered coherence between various brain regions and reductions in theta-gamma phase-amplitude coupling (PAC) [9, 24, 35–39]. Many of these alterations have been shown to correlate with the severity of AD symptoms [40].

We therefore have used a seeding approach at the LC nuclei of the tau transgenic mouse strain, tauP301L, chronically equipped with depth multichannel LFP/EEG recording electrodes in key brain locations, to investigate functional network dynamics in relatively distant areas from the seeding location [41]. The tauP301L mouse model was chosen for this study due to its reasonably slow pathology: neurofibrillary tangles are seen from around 4.5 months of age, while neurodegeneration and astrogliosis occur from around 10 months of age [15]. This large window of opportunity allowed us to study any possible functional changes, in the relative absence of severe endogenous tau pathology and neurodegeneration. A recent study, by [30], used a similar approach, using PS19 mice, another tau line with reasonably slow pathology, in order to disentangle endogenous and exogenous tau pathologies. In addition, we investigated whether the lack of tau pathology in the entorhinal cortex and the hippocampus seen in the previously mentioned LC seeding model [30] was associated with alterations in functional connectivity measures to support or challenge the view that pathology spread to an area is necessary to elicit functional impairments in that area.

## 2. Materials and Methods

### 2.1. Animals and Surgical Procedures

All experiments were conducted in strict accordance with the guidelines of the Association for Assessment and Accreditation of Laboratory Animal Care International (AAALAC) and with the European Council Directive of 24 November 1986 (86/609/EEC). All protocols were approved by the local Institutional Animal Care and Use Committee. For this study, 16 male transgenic tauP301L mice, expressing the longest human tau isoform, were used for surgery at the age of 3 months [42]. All mice were implanted with a chip for identification using the Animal Inventory and Weighing (AIW) system. For this study, all animals were housed in full-view Plexiglas® cages (25 cm × 33 cm × 18 cm), in IVC (individually ventilated cage) racks, within a sound-attenuated chamber with controlled environmental conditions: 22 ± 2°C ambient temperature, 60% relative humidity; 12 : 12 light-dark cycle (lights on at 1900 hours, lights off at 0700 hours), light intensity ~100 lux, and food and water available ad libitum.

### 2.2. Histology Evaluating the Accuracy of the LC Injection Site using Evans Blue Dye

Adult male C57/BL6 mice were deeply anesthetized with isoflurane during the surgical procedures, confirmed by observation of breathing frequency and responsiveness to toe pinching. Anesthesia was induced with 5% isoflurane and maintained with 2% isoflurane while body temperature was maintained at 37°C with a heating pad. To verify correct targeting of the LC site, immunohistochemistry (IHC) technique was performed using Evans blue dye. The LC was stereotaxically (David Kopf Instruments) injected with 1% Toluidine blue solved in distillated water, and a Hamilton syringe of 10 microliters connected to a syringe pump was used to inject a volume of 5 microliters/10 minutes at the stereotaxic mouse brain atlas coordinates of AP: -5.4 mm from the bregma, ML: +1.3 mm, and DV: -3.6 mm [43]. Afterwards, the brains were removed and trimmed after fixation with a mouse brain trimmer. Trimmed specimens were processed, sectioned in serial sequential levels, stained, and examined.

### 2.3. Tau-Seeding Procedure

**Figure 1:**
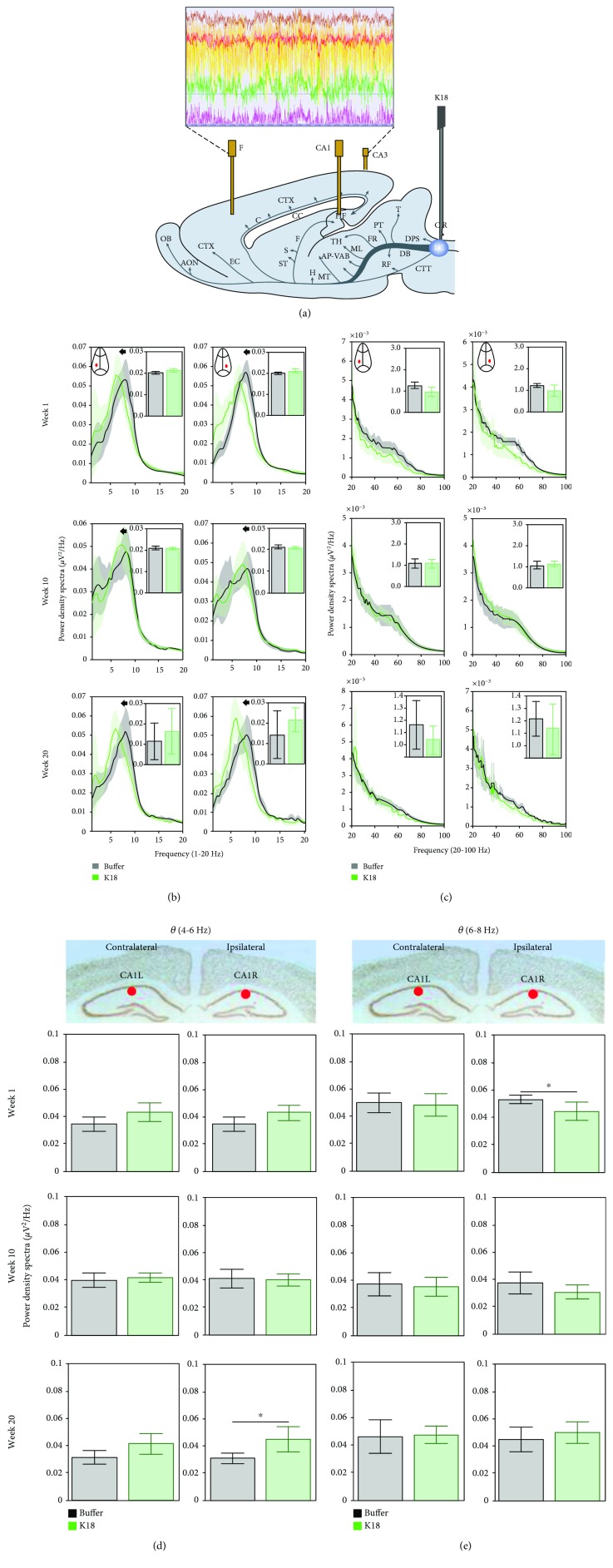
(a) Scheme showing the placement of recording electrodes and a cannula for injection of K18 in the locus coeruleus. (b, c) Relative power spectra in frequency (1-20 Hz (b) and 20-100 Hz (c)) for the CA1R and CA1L electrodes for buffer- (black, *n* = 7) and K18- (green, *n* = 8) injected mice, at recording weeks 1, 10, and 20. Insets indicate total relative power with no significance between group differences (two-sample *t*-test) at 1-20 Hz and 20-100 Hz in b1 and b2, respectively. (d, e) Relative power spectra in CA1R and CA1L in low (4-6 Hz (d)) and high (6-8 Hz (e)) theta oscillations at recording weeks 1, 10, and 20. Data are presented as mean (across animals) values (and 95% CI). Horizontal lines above bar plots with asterisks indicate the presence of significant difference between buffer- and K18-injected animals (two-sample *t*-test; ^∗^*p* value < 0.05).

At 3 months of age, animals underwent surgery for tau seeding and implantation of electrodes for local field recordings. Animals were anaesthetized with isoflurane, mounted in a stereotaxic frame, with the incisor bar 5 mm beneath the center of the ear bar. Following this, the animals were unilaterally injected with either preformed synthetic tau K18 fibrils (*n* = 8) or buffer (*n* = 8) into the right-side LC [16, 30, 40]. 25 *μ*g of K18, in a volume of 5 *μ*L, or 5 *μ*L of buffer was injected at a rate of 1 *μ*L/min, using a 5 *μ*L Hamilton syringe (Hamilton Company), after which the needle was left in place for 5 minutes before being gently withdrawn. Afterwards, animals were stereotaxically equipped with 6 stainless steel recording electrodes (Figure 1(a)) in the frontal cortex (FC) (AP + 2 mm, *L* ± 1.4 mm), CA1 (AP − 1.7 mm, *L* ± 1.5 mm, and ventral ± 1.8 mm), and CA3 (AP − 2.8 mm, *L* ± 3.2 mm, and ventral ± 3.5 mm) [24]. All electrodes were referenced to a ground screw electrode, placed above the midline of the cerebellum. Electrodes were connected to a pin (Future Electronics: 0672-2-15-15-30-27-10-0) with a small insert (track pins; DataFlex: TRP-1558-0000) and were inserted into a 10-hole connector, which was carefully fixed to the skull with dental cement.

### 2.4. Experimental Design, Recording, and Analysis

After the one-week recovery period, animals were allowed another week to adaptation to the recording conditions. Body weight was measured prior to surgery and prior to each recording session, in order to monitor for rapid, pathological weight loss and to allow investigation into potential differences in weight gain between the two treatment groups. Due to variability in body weight prior to surgery, data were analyzed as the percentage of body weight change from baseline (presurgery). Core body temperature was taken on one occasion, using a rectal temperature probe to determine whether there were any deviations in this measure between buffer- and K18-injected animals. EEG recordings were taken for 3 hours, once a week, for 20 consecutive weeks. EEG recordings were performed during the dark phase of the circadian cycle, under vigilance-controlled wake, as described elsewhere [7]. Recordings were taken in the animal's home cages, fitted with a removable insert, and placed inside environmentally controlled, sound-attenuated Faraday cages. Motor activity was measured by a pair of passive infrared (PIR) detectors located above every recording cage. Motion levels were analyzed from the envelope of activity from both PIR detectors. Only continuous, waking, artefact-free epochs were used in the analysis. A notch finite impulse response (FIR) filter at 50 Hz was used to filter out possible noise caused by the main power line interference. EEGs were recorded at a sampling rate of 512 Hz using a BioSemi ActiveTwo system (BioSemi, Amsterdam, Netherlands), digitized with a resolution of 24 bits.

### 2.5. EEG Spectra

Analysis was performed using MATLAB analysis modules. Spectral power density was calculated using Welch's method with the Hanning window function and a block size of 512 data points, giving a resolution of 1.0 Hz. Power was expressed as relative power for each frequency over 1-256 Hz. Average relative power in each frequency bin of each location was averaged across animals for the buffer- and K18-injected groups to obtain the spectrum relative to the total power spectrum. For the sake of clarity in presenting this spectral data, graphs only show the frequency range between 1 and 20 Hz and inset plots from 20 to 100 Hz.

### 2.6. Phase-Amplitude Cross-Frequency Coupling

To estimate whether high-frequency EEG amplitudes are modulated by low-frequency phase variations for the same (intrasite electrode) or different (intersite electrode) signals, phase-amplitude coupling (PAC) was calculated using the algorithm based on the modulation index (MI) [24, 44, 45]. MI is estimated as a mean (along time *t*) absolute value of the signal *z*(*t*) = *A*_H_(*t*) × exp(*i* × *φ*_*L*_(*t*)), $i=\sqrt{-1}$, using the instantaneous phase *φ*_L_(*t*) derived via the Hilbert transform from the narrow bandpass-filtered signal around the low frequency *f*_L_, and instantaneous amplitude envelope *A*_H_(*t*) derived via the Hilbert transform from the narrow bandpass-filtered signal around the high frequency *f*_H_. For PAC estimation, *f*_L_ was varied in a 2-12 Hz interval with a step of 2 Hz and *f*_H_ taken from a 10-200 Hz interval with a step of 5 Hz was considered.

Modulation of *A*_H_(*t*) in relation to phases *φ*_L_(*t*) could also be represented graphically, if one plots for every phase *φ* ∈ [−2*π*, 2*π*) of the narrow bandpass-filtered signal around the low frequency *f*_L_ an averaged amplitude value *A* (taken for time points, when the correspondent phase is equal to *φ*) from the narrow bandpass-filtered signal around the high frequency *f*_H_. Larger variations in amplitude of the obtained near “sinusoidal” curve correspond to higher values of MI. In addition, it becomes possible to estimate the *phase shift* for the obtained curve. This phase shift value could be further used for comparison between groups or experimental conditions.

### 2.7. Coherence

In order to describe interconnectivity between pairs of EEG electrodes, coherence index was measured, which describes the level of connectivity as a value in interval [0, 1] (where 1 corresponds to complete perfect relation) for each frequency band *f*. *Coherence* is estimated as Coh(*f*) = |*S*_AB_(*f*)|^2^/(*S*_AA_(*f*)*S*_BB_(*f*)), where *S*_AB_ is the cross-spectrum between signals A and B from two different electrodes, *S*_AA_ is the autospectrum of signal A, and *S*_BB_ is the autospectrum of signal B. Such a pairwise estimation of coherence between all electrodes leads to a network, which could be represented as a graph with electrodes as nodes and coherence values as distances between nodes (weights of edges). To estimate the level of interaction in such a brain network, the global efficiency index (Eglob) was estimated as a mean of inverse shortest distances along all pairs of electrodes [46]. Eglob could be estimated either for a particular frequency *f* or for a frequency band. In the latter case, mean (along all frequencies in a band) coherence values between pairs of electrodes were used as distances between nodes.

Coherence analysis allows assessment of pairwise synchronization of LFP/EEG signals to shed more light onto the interaction between different brain networks. Highly coherent oscillations between two structures can occur because they are functionally connected or because they share a common input. To exclude indirect relation though other areas where we have recording from, the measure of *partial coherence* was used [47]. Partial coherence between signals A and B is estimated as $M_{\text{AB}}/\sqrt{M_{\text{AA}}M_{\text{BB}}}$, where *M*_AB_ is a minor of a spectral matrix (matrix of spectra and cross-spectra between all pairs of electrodes) with the A-th row and B-th column removed.

To further add to the assessment of the information flow in a brain network, *extended partial directed coherence* was used [48]. It provides strength of causal *directional* coupling between pairs of electrodes, excluding relations due to the paths through other areas where we have recordings from.

## 3. Statistical Analysis

**Figure 2:**
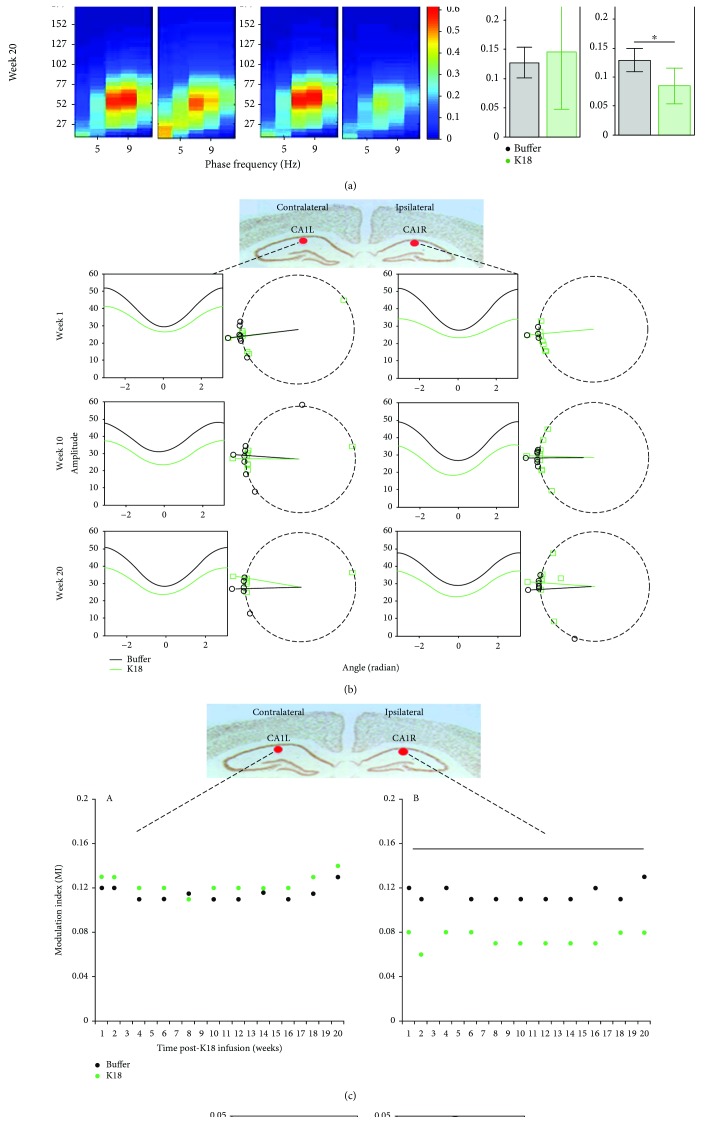
(a) Heat maps showing the mean phase-amplitude coupling (PAC) modulation index at the CA1L and CA1R electrodes for buffer- (black, *n* = 7) and K18- (green, *n* = 8) injected mice, at recording weeks 1, 10, and 20. As shown by the color scale, “hotter” colors indicate high coupling values while “colder” colors indicate low or no coupling. Bar graphs showing the mean (across animals) theta-gamma PAC (with 95% CI) at the CA1L and CA1R electrodes for buffer- (black, *n* = 7) and K18- (green, *n* = 8) injected mice, at recording weeks 1, 10, and 20. These means along animals' PAC values are calculated as the average PAC for the window of phase frequency: 3.5–12.5 Hz, and amplitude frequency: 32–100 Hz, to focus on theta-gamma PAC. Horizontal lines above the bar plots with asterisks indicate the presence of significant difference between buffer- and K18-injected animals (two-sample *t*-test; ^∗^*p* value < 0.05 and ^∗∗^*p* value < 0.01). (b) Averaged across animals' variations in gamma amplitude (vertical axes) as a function of theta phases (horizontal axes) obtained from the electrodes implanted in CA1L and CA1R for the weeks 1, 10, and 20 postadministration of the buffer and K18. Right plots show estimated phase shifts in obtained oscillations for each animal (shown as dots) and condition (buffer (black) and K18 (green) injected). Radii show circular mean values for buffer- and K18-injected groups of animals. No significant difference in means between groups across all time points was found with the Watson-Williams test. (c) Scatter graphs show mean theta-gamma PAC at the contralateral (CA1L) and ipsilateral (CA1R) CA1 regions of the K18 injection site, for all recording weeks, demonstrating changes in PAC over time, for the buffer-injected (black) and K18-injected (green) groups. Time intervals with significant differences (*p* < 0.05, two-sample *t*-test) between buffer-injected and K18-injected animals are shown by a horizontal line. (d) Bar charts quantifying the mean PAC in frontal electrodes for buffer- (black) and K18- (green) injected animals. Note that no significant difference (two-sample *t*-test) was observed between the study groups. Data are presented as mean values (with 95% CI).

Results for described EEG metrics and for groups of buffer- and K18-injected animals are presented as mean values with 95% confidence intervals (CI). Between-group difference in means was assessed using the two-sample *t*-test, and in case of significance, it is indicated by asterisks on box plots (^∗^*p* value < 0.05, ^∗∗^*p* value < 0.01). For phase shift data (see Figure 2(b)), which are circular in nature, differences in means were tested using the Watson-Williams test. In case of significance, asterisks provide a level of significance (^∗^*p* value < 0.05, ^∗∗^*p* value < 0.01). Difference in means between groups was considered significant, if the *p* value is below 0.05.

## 4. Results

### 4.1. Accuracy of LC Injection Sites

**Figure 3:**
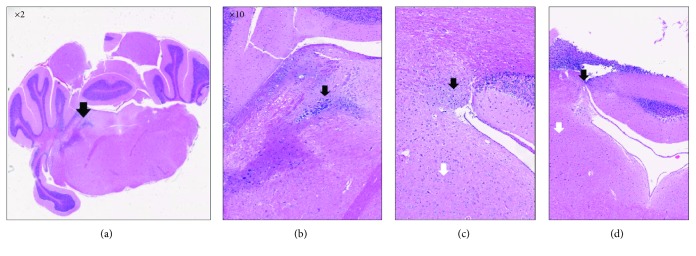
The image in (a) is an image from the mouse brain atlas depicted with black arrows in the LC region from a sagittal view. Areas of interest: microscopic images of coronal views of the neurons of the LC (a, b) and the Purkinje/molecular layer neurons around the LC (c, d).

The results shown in Figure 3 indicate that the Evans blue dye was successfully injected into the LC area. Coronal histological images from mouse brains show that the blue dye was present exactly in the neurons of LC (Figures 3(a) and 3(b)) and in the Purkinje/molecular layer neurons around the LC (Figures 3(c) and 3(d)), which may suggest that the fast-blue ink may have been washed out from LC neurons.

### 4.2. No Changes in Mean Locomotor Activity, Body Weight, Body Temperature, and Food Intake Were Observed between K18- and Buffer-Injected Mice

**Figure 4:**
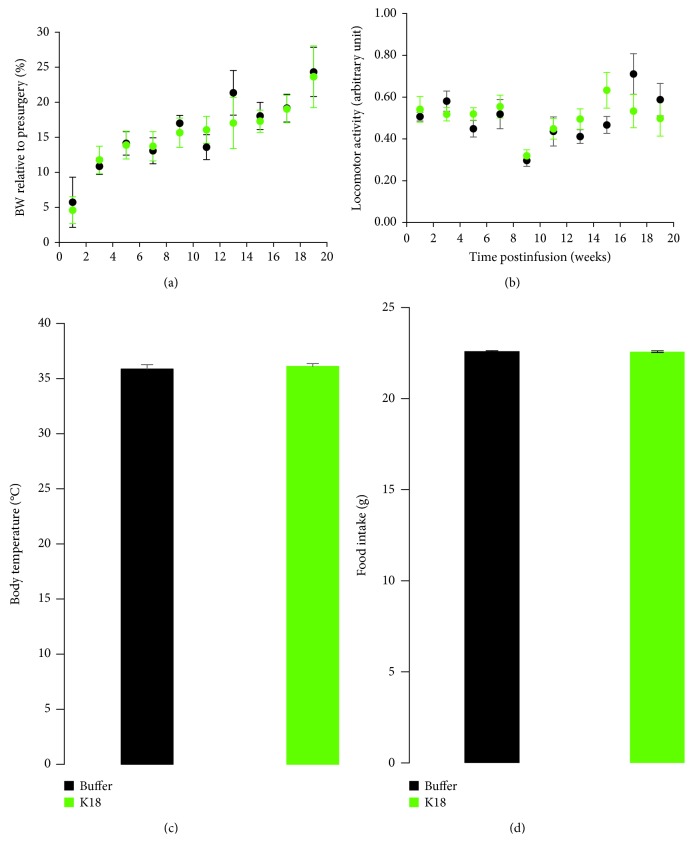
Mean locomotor activity, body weight, body temperature, and food intake in buffer- (black, *n* = 7) and K18- (green, *n* = 8) injected mice. (a) Body weight relative to presurgery and (b) locomotor activity were monitored daily prior and during EEG recording sessions, respectively. (c) Body temperature was measured in the middle of the study, and (d) food intake was measured daily during the first week postinfusion of K18 and buffer in the LC area. No changes in the mean activity levels, body weight, body temperature, and food intake were observed between the study groups. Data are expressed as mean ± sem.

The body weight and locomotor activity were longitudinally monitored throughout the study. There were no significant differences (two-sample *t*-test) in average activity levels and body weight between K18- and buffer-treated mice (Figures 4(a) and 4(b)). Body temperature and food intake were assessed in the same group of animals, and both measures remain similar between the study groups at the measurement time points (Figures 4(c) and 4(d)).

### 4.3. Tau-Seeded Animals Demonstrate Progressive Shift in Theta Relative Power Spectra in the CA1 Region of the Hippocampus Ipsilateral and Contralateral to the Injection Site

We present the effects of tau seeding in the LC on the oscillatory activity of the ipsilateral and contralateral hippocampal CA1 regions as shown by a relative power spectral density of over 1-20 Hz (plots in Figure 1(b)) and 20-100 Hz (plots in Figure 1(c)). Seeding of K18 aggregates in the LC (Figure 1(a)) was shown to cause a significant leftward shift in the theta frequency activity in the contralateral and ipsilateral CA1L/R regions at recording weeks 1, 10, and 20 as compared to buffer-injected animals (*p* < 0.05, the two-sample *t*-test for the center of mass in the theta band; large plots in Figure 1(b)), while the relative theta power did not change (Figure 1(b), insets with bar plots). At recording week 1, this progresses, with an additional reduction (*p* < 0.05, two-sample *t*-test) in the high gamma oscillations (*γ*1 50-80 Hz) (Figure 1(c)). Quantification of the relative power of over 1-20 Hz did not reveal a major difference between groups (Figures 1(d) and 1(e), inset bars), whereas a significant (*p* < 0.05, two-sample *t*-test) increase in slow theta (4-6 Hz) was observed in the final recording week 20.

### 4.4. Tau-Seeded Animals Present with Early, Severe Reductions in Intra-Theta-Gamma Phase-Amplitude Coupling in the CA1 Region Ipsilateral to the Injection Site

Recent evidence highlights the functional relevance of temporal relationships between superimposed network oscillations during information processing in the brain [49–51]. We therefore estimated the strength of cross-frequency coupling between the phase of slow and the amplitude of fast oscillations in different recording sites. Mean (across animals) PAC values at the contralateral CA1L and ipsilateral CA1R regions are qualitatively shown in the form of comodulation heat maps for buffer- and K18-injected mice (Figure 2(a), heat maps), at recording weeks 1, 10, and 20. Of note, we show figures of these 3 weeks as a similar pattern was observed throughout the recording weeks. As shown in Figure 2(a), left bar plots, buffer-injected animals demonstrate equivalently (no statistical difference, two-sample test) high phase-amplitude coupling in both ipsilateral CA1R and contralateral CA1L regions, at recording weeks 1, 10, and 20. This high coupling peaks around a phase frequency of 7.5 Hz and amplitude frequency of around 52 Hz, in the theta-gamma range (see Figure 2(a), left comodulation heat maps).

Conversely, Figure 2(a), right comodulation heat maps, demonstrates reduced PAC in the ipsilateral CA1R of K18-injected animals at recording week 1, which seems to worsen at recording week 10 and persists during recording sessions up to week 20. PAC comodulation heat maps in the contralateral CA1L of K18-injected animals also seem to be slightly impaired, but to a much lesser extent (Figure 2(a)). Quantification in the form of bar charts at recording weeks 1, 10, and 20 showed a significantly (*p* < 0.05, two-sample *t*-test) reduced mean theta-gamma PAC in the ipsilateral CA1R region in the K18-injected group as compared to the buffer-injected group (Figure 2(a), right bar plots).

To further investigate whether the pattern of phase-amplitude coupling is different between groups of animals, we sorted the gamma amplitudes by the theta phases and computed the mean gamma amplitude for each radian wide bin (Figure 2(b)) at weeks 1, 10, and 20 postadministration of the buffer and K18. Our results confirm what we already saw using PAC, i.e., that gamma band activity could be modulated by the phase of theta activity. At the same time, no phase shift was revealed in all comparisons (Watson-Williams test, Figure 2(b), right plots). However, the averaged amplitude of gamma oscillation for all phases of theta oscillation was reduced in K18-treated animals throughout the recording session (Figure 2(b)).

### 4.5. Theta-Gamma PAC Steadily Deteriorates in the CA1 Region of the Hippocampus Ipsilateral to the Injection Site throughout the 20-Week Recording Period

Figure 2(c) displays the mean theta-gamma PAC at the ipsilateral and contralateral CA1 for all recording weeks, from 1 to 20, to show changes in the trajectory of the PAC strength over time in the buffer-injected and K18-injected groups (Figure 2(c), A and B, respectively). In the buffer-injected group, the mean theta-gamma PAC is almost the same as those in both the ipsilateral CA1R and contralateral CA1L regions from recording week 1 to recording week 20 (no significant difference, two-sample *t*-test applied for each week of data separately) and stays at a high mean value of around 0.12. However, in the K18-injected group, the mean theta-gamma PAC was greatly reduced throughout the study at the ipsilateral CA1R region (*p* < 0.05, two-sample *t*-test applied for each week data separately), staying at a mean value of around 0.07 (Figure 2(c), B). There were no major changes (no significant group difference, two-sample *t*-test) in the PAC strength in cortical regions between the study groups, through the recording sessions (Figure 2(d))

### 4.6. Intersite PAC Indices between Electrodes Demonstrate Significant Reductions in Phase-Amplitude Coupling between Contralateral and Ipsilateral CA1 Theta-Gamma Oscillations, in Tau-Seeded Animals

**Figure 5:**
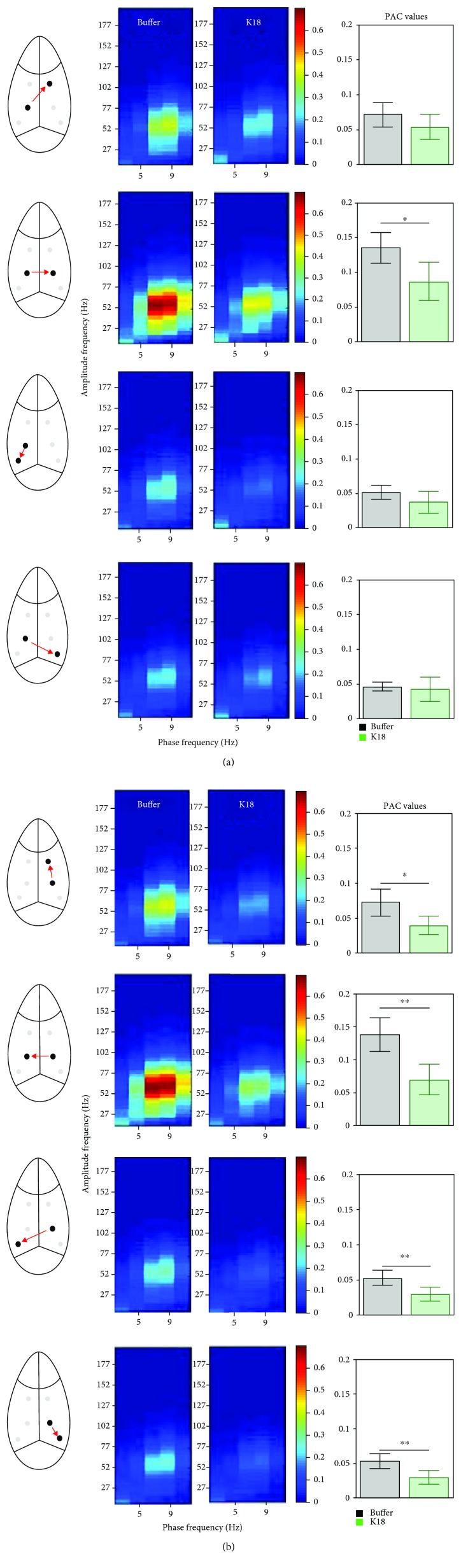
Heat maps showing the mean phase-amplitude coupling (PAC) between contralateral CA1L and ipsilateral CA1R electrodes for buffer- (left heat maps in each frame) and K18- (right heat maps in each frame) injected animals at recording week 1 (buffer *n* = 7, K18 *n* = 8). (a) CA1L > CA1R represents the strength of PAC between theta oscillations (phase) from CA1L and gamma oscillations (amplitude) at CA1R, while (b) CA1R > CA1L represents the strength of PAC between theta oscillations from CA1R and gamma oscillations at CA1L. Bar charts quantifying the mean PAC between the stated electrodes (shown with 95% CI) for buffer- (black) and K18- (green) injected animals. These mean PAC values are calculated as the average PAC for the window of phase frequency: 3.5–11 Hz, and amplitude frequency: 32–100 Hz, to focus on theta-gamma PAC. Horizontal lines above the bar plots with asterisks indicate the presence of significant difference between buffer- and K18-injected animals (two-sample *t*-test; ^∗^*p* value < 0.05 and ^∗∗^*p* value < 0.01).

Theta-gamma PAC not only is an important mechanism underlying synaptic plasticity in one region of the brain but can also facilitate communication and functional connectivity between distant brain regions. Unlike the previously mentioned intrasite PAC, intersite PAC analyses characterize coupling of oscillations in one brain region with oscillations in another brain region, which gives hints into the directionality of these interactions. Therefore, we questioned how the phase of theta oscillations in one brain region may modulate the amplitude of gamma oscillations in a different region. We investigated theta-gamma PAC between contralateral CA1L and ipsilateral CA1R electrodes, as shown in Figure 5. In the buffer-injected group, as shown in Figures 5(a) and 5(b), left heat maps, there was high theta-gamma PAC from the contralateral CA1L electrode to the ipsilateral CA1R electrode and vice versa. It is also of note that mean CA1L > CA1R PAC and mean CA1R > CA1L PAC were almost equal. However, in the K18-injected group, as shown in Figures 5(a) and 5(b), right heat maps, PAC between CA1L and CA1R was reduced in both directions as compared to that in the buffer group, although this reduction seems slightly greater in the CA1R > CA1L direction (Figures 5(a) and 5(b), bar plots). The mean phase of theta frequency oscillations in the frontal and CA3 regions and the amplitude of CA1 gamma frequency were also significantly reduced (*p* < 0.05, two-sample *t*-test, Figure 5(b), bar plots).

### 4.7. Tau Seeding Alters the Functional Cortical Network

**Figure 6:**
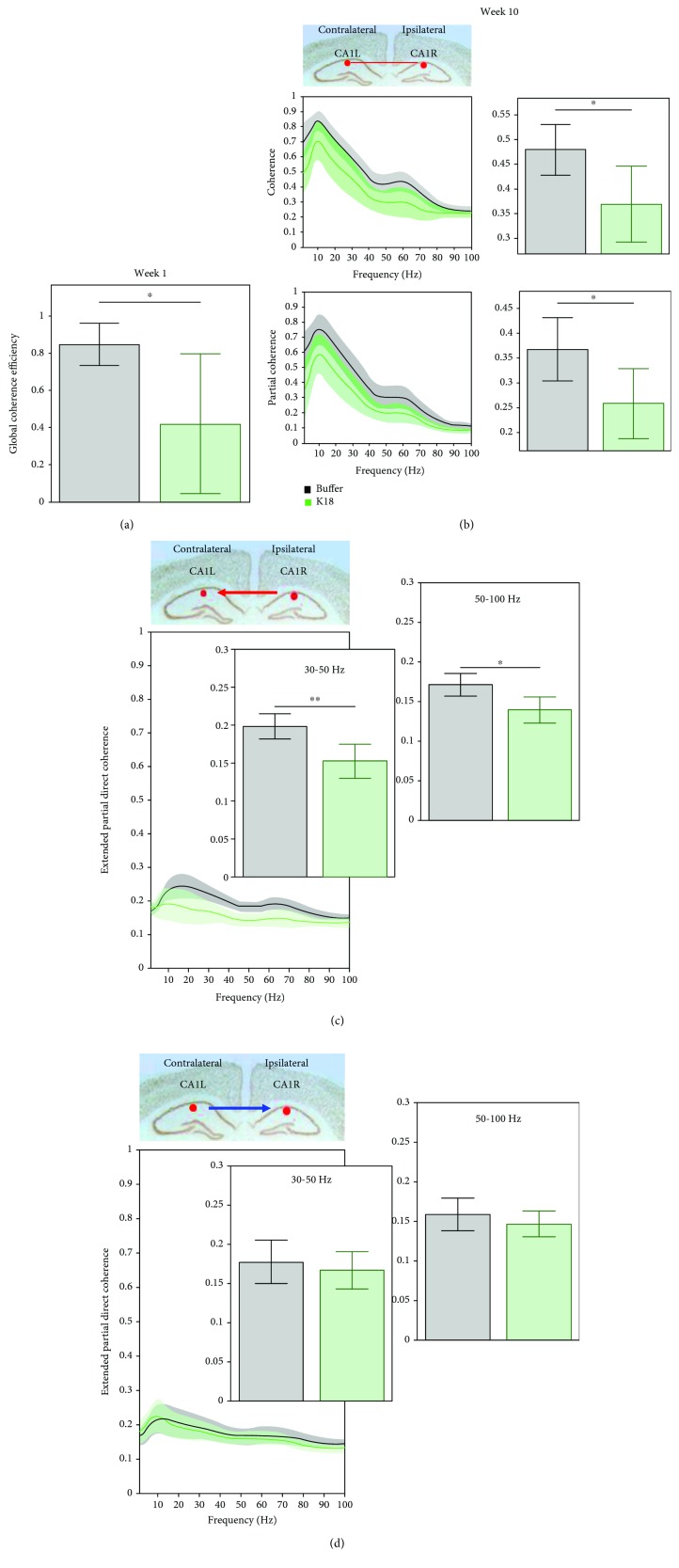
(a) Graphs showing the mean (across animals) global coherence efficiency (with 95% CI) for buffer-injected (black) and K18-injected (green) mice, at week 1 (buffer *n* = 7, K18 *n* = 8). Horizontal lines above bar plots with asterisks indicate the presence of significant difference between buffer- and K18-injected animals (two-sample *t*-test: ^∗^*p* value < 0.05). (b) The top line graph shows the mean (across animals) coherence values (with 95% CI) between CA1L and CA1R electrodes for buffer-injected (black) and K18-injected (green) mice, at week 10 for the frequency range 0-100 Hz. On the right-hand side, bar charts show the mean (across animals) coherence (with 95% CI) for both groups for the frequency range of interest. Bottom line graphs show the mean (across animals) partial coherence values (with 95% CI) between the same electrodes. On the right-hand side, bar charts show the mean partial coherence (with 95% CI) for both groups for the same frequency range, 0-100 Hz. Horizontal lines above bar plots with asterisks indicate the presence of significant difference between buffer- and K18-injected animals (two-sample *t*-test; ^∗^*p* value < 0.05). (c) Graphs showing the mean (across animals) extended partial directed coherence (with 95% CI) from CA1R to CA1L and (d) CA1L to CA1R for buffer-injected (black) and K18-injected (green) mice, at recording week 10. On the left-hand side, these data are presented in the form of a line graph, showing mean (across animal) extended partial directed coherence as a function of frequency (with 95% CI) for both groups for the frequency range 0-100 Hz. On the right-hand side, bar charts show the mean (across animals) extended partial directed coherence (with 95% CI) for both groups for frequency ranges 30-50 and 50-100 Hz. Horizontal lines above the bar plots with asterisks indicate the presence of significant difference between buffer- and K18-injected animals (two-sample *t*-test; ^∗^*p* value < 0.05 and ^∗∗^*p* value < 0.01).

Coherence characterizes synchronous oscillations in different networks and deduces functional coupling among these networks. As shown in Figure 6(a), there was an early relatively weak (*p* < 0.05, two-sample *t*-test) mean global coherence efficiency in K18-injected animals estimated for a whole network of our recordings, as compared to buffer-injected animals. This reduction in a whole network coherence was primarily driven by the coherence between the CA1L and CA1R recording sites. CA1L-CA1R coherence function, which peaks in the theta and high gamma frequency bands, shows generalized decrease (*p* < 0.05, two-sample *t*-test) in the 4-70 Hz frequency range at recording week 10 in the K18-injected group, as shown by the accompanying bar charts (Figure 6(b), upper graph and bar chart).

Partial coherence analysis is similar to the previously mentioned coherence analysis but removes the influence of indirect connections, in order to focus on robust connections and remove the possible effects of volume conduction. Due to this removal, partial coherence is lower in comparison to coherence, as it can also be seen for CA1L-CA1R results for week 10 (Figure 6(b), lower graph and bar chart). Mean partial coherence levels were significantly (*p* < 0.05, two-sample *t*-test) decreased between CA1L and CA1R electrode pairs in the 4-70 Hz frequency range in the K18-injected group, as compared to the buffer-injected group.

Partial directed coherence, which is an extension of partial coherence, probes time lags in signals to investigate the directionality of these coherent interactions and focuses on causal functional relationships. As shown in Figure 6(c), at recording week 10, there were changes in mean extended partial directed coherence between CA1L-CA3R electrode pairs. A consistent decrease in the extended PDC levels was observed for low and high gamma oscillations from CA1R > CA1L (*p* < 0.05, two-sample *t*-test, Figure 6(c)). However, there seems to be no significant difference (two-sample *t*-test) in the level of directional interactions from CA1L > CA1R (Figure 6(d)) or within the FL-FR and CA3L-CA3R (data not shown) electrode pairs.

## 5. Discussion

K18 seeding in the brainstem LC elicited early robust alterations in distant hippocampal functional networks, and as seen before, the pathology spread from the deeper LC seeding area to the hippocampus and frontal cortex was not observed [30].

While no current tau transgenic mouse models of AD fully recreate the spatiotemporal progression of tau in humans, they have taught us a great deal about the nature of tau pathology and its direct involvement in AD. Human tauopathies and numerous tau transgenic mouse models have demonstrated that tau pathology can cause neurodegeneration and cognitive impairment in the absence of A*β* [15, 16, 52]. However, various studies have also shown that tau pathology causes functional alterations in affected neurons aside from neuronal injury. One such study by [16] found that suppressing the human mutant tau transgene in the aforementioned rTg4510 tau transgenic mouse line restored cognitive function and prevented further neuronal loss, while aggregation of tau into NFTs continued. This recovery of cognitive function implied that these early impairments were due to “reversible neuronal dysfunction” and not necessarily due to neurodegeneration. These results also suggest that the pathological form of tau may in fact be the soluble tau oligomers, rather than the insoluble NFTs, a theory echoed by others in the field [53]. A seminal study by [33] demonstrated that neurons with intracellular tau show reduced activity and a relatively small number of pathological neurons can affect the activity of whole networks. From the various electrophysiological alterations seen in our mouse model, we expected that the cause of these alterations would be due to the rapid spread of tau pathology from the seeded locus coeruleus to the ipsilateral hippocampus and its direct effects on this region. However, surprisingly, no tau pathology was seen in the hippocampus of a previous LC tau-seeding model [30]. A possible explanation suggests that this previous result may be due to strain-specific effects; however, a pilot study revealed no tau deposits at the level of the CA1 (data not shown). One possible explanation for this lack of tau pathology in the hippocampus could be due to the relatively short time frame of the study and that the spread of tau pathology to the hippocampus may occur in a given time. Another possibility is that tau pathology could have spread to the hippocampus but, by 20 weeks postinjection, was effectively removed by different clearance systems in the brain. However, there is also another possibility that has been brought up in a recent paper [54], in which the authors suggest that the spread of tau beyond the earliest Braak stages could be accelerated by increasing concentrations of A*β*, through “cross-seeding” of tau by A*β* [25]. Therefore, it could be that the lack of high levels of A*β* in the hippocampus and frontal cortex prevents rapid spread of tau pathology to these areas. One way of investigating this could be to seed tau pathology in the LC of an amyloid transgenic mouse model and investigate the subsequent pathology. One caveat to this however is the possibility that the lack of mutant tau transgenes in these animals could prevent effective tau seeding in these animals. Further work down this line of investigation could be rewarding, as any AD model is limited in its usefulness without the presence of both tau and amyloid pathologies. Regardless, the robust functional alterations seen in the hippocampus in our model in the absence of tau pathology challenge the classic prion hypothesis that the spread of tau pathology to areas of the brain may not be the sole causal factor that results in functional impairments in these areas [21].

To understand the causes of the various functional alterations demonstrated in the LC seeding model, it is salient to first look at them individually, as there is nothing to imply that they are resulting from a single common factor.

### 5.1. Hippocampal Spectral Changes Seem to Demonstrate Altered Neural Network Activity

Firstly, the most notable change in hippocampal power spectra is the early entrainment shift in relative power oscillations of a range of frequencies at the CA1 contralateral and ipsilateral to the injection site of K18. At the first week post-K18 injection, there was a shift in the peak theta frequency oscillation towards slow rhythm and reduction in the peak gamma frequency oscillations. Furthermore, at 20 weeks post-K18 injection, the sustained entrainment shifts in relative power seem to slightly reflect the “slowing” of the EEG, which refers to a shift in the power spectrum due to decreased high-frequency oscillations and subsequently increased low-frequency oscillations as seen in AD [9]. Therefore, our observation in the CA1R of tau-seeded animals may be indicative of the beginnings of this EEG slowing. The cause of this slowing in AD is currently not certain, but earlier works have shown that combined cholinergic and monoaminergic blockade in rats can effectively recreate this slowing and also results in severe cognitive impairments [7, 55, 56]. It is highly possible that tau pathology in the noradrenergic LC and nearby cholinergic nuclei could result in reduced noradrenergic and cholinergic signalling to the hippocampus, resulting in this slowing. Clinical evidence has demonstrated that there is an upregulation of beta-2 adrenoceptors at the hippocampus and frontal cortex of AD patients, suggesting a compensation for reduced noradrenergic input, while muscarinic M1 receptor densities have been shown to be largely unchanged, suggesting reduced cholinergic innervation as the cause of loss of cholinergic signalling in AD [57]. The maintenance of theta power in this region seems to be extremely fortuitous, as the importance of hippocampal theta rhythms in cognition and short-term memory has been well documented [58]. It has also been suggested that impairments of hippocampal theta through functional means may be as detrimental to mental health as impairment of hippocampal theta through structural damage [59]. Due to the generally unidirectional nature of information flow in the hippocampal trisynaptic circuit, one of the main inputs to the CA1 is the CA3 region. No differences were found in the ipsilateral CA3 power spectra between K18- and buffer-injected animals. However, there seems to be a slight, nonsignificant decrease in slow and fast theta power at this electrode for the 10-20-week period. The power spectra of the contralateral CA1L show no significant differences in K18-injected animals in any frequency band, at any week, and show no notable changes either. However, interestingly, the power spectra of the contralateral CA3L region show nonsignificant changes in theta power. Although the hippocampi of both hemispheres are separate, they are strongly interconnected through the hippocampal commissure, and while there is a weak commissural connection between CA1 regions, one of the main origins of hippocampal commissural fibers is CA3 pyramidal cells [60]. These commissural fibers innervate the CA1, CA2, and CA3 regions of the contralateral hippocampus. Therefore, it is entirely possible that any change in the theta power at the CA3L region is maintaining theta power at the CA1R through commissural fibers.

### 5.2. Reduced Theta-Gamma PAC at the Ipsilateral CA1 Region Could Result in Impaired Synaptic Plasticity in This Region

Theta-gamma PAC has been widely researched due to its possible roles in learning and memory. PAC strength has been shown to correlate with cognitive performance [48], and as demonstrated in numerous in vitro electrophysiology protocols, gamma frequency stimulation bursts repeated at theta frequency effectively induce long-term potentiation, a type of synaptic plasticity in the hippocampal CA1 area [61, 62]. Impaired theta-gamma PAC has been demonstrated prior to A*β* accumulation in an amyloid mouse model AD [36] and in the tau-seeding model [24] and has been suggested to be a possible early functional biomarker of AD [63]. Here, K18 seeding in the LC resulted in reduced theta-gamma PAC at the ipsilateral CA1R region, from 1 week postinjection, and persisted throughout the 20-week recording period. This significant early impairment occurs in the CA1R of these animals prior to a significant reduction in gamma power and in the absence of changes in the phase angle or theta power. Additionally, theta-gamma PAC at the contralateral CA1 was shown to steadily deteriorate throughout this 20-week recording period. Similarly, this reduction in theta-gamma PAC in the contralateral CA1L occurs in the absence of significant changes in theta or gamma power. One possible explanation for this is due to a reduction in cholinergic signalling from affected cholinergic nuclei in the brainstem. It has been shown that reduced cholinergic signalling, through systemic administration of the muscarinic antagonist scopolamine, causes impaired theta-gamma PAC in the entorhinal cortex of freely moving rats [64]. Intracellular tau has been shown to reduce the firing rates of neurons prior to neurodegeneration [32], so developing tau pathology in brainstem cholinergic nuclei could reduce cholinergic signalling as a robust reduction in the number of choline acetyltransferase- (ChAT-) immunopositive cholinergic neurons that has been previously seen in the medial septum of a tau transgenic mouse model [65].

In Alzheimer's disease, reduced cholinergic signalling has been established as a cause of cognitive impairment and has been attributed to reduced cholinergic innervation [54]. The deterioration of theta-gamma PAC in the contralateral CA1L could also be explained by this, as tau pathology in the brainstem spreading to the contralateral side would result in increasing numbers of affected cholinergic neurons on that side and therefore decreasing signalling. While the cause of impaired theta-gamma PAC in the aforementioned amyloid model was likely resulting from the local electrophysiological alterations caused by increasing levels of A*β* in the hippocampus, in the context of AD, the combination of these two different pathologies could conceivably result in an even greater theta-gamma PAC impairment.

### 5.3. Bidirectional Theta-Gamma PAC Impairments between CA1L and CA1R in Seeded Animals

Theta-gamma PAC has also been suggested to facilitate communication between distant brain regions [66]. Intersite PAC analysis was used to investigate changes in the coupling of oscillations between contralateral and ipsilateral CA1 regions. This allows understanding of functional changes in the dynamics of this network and allows possible further insight into the nature of localized PAC changes at these regions. At recording week 1, there was significantly reduced coupling of contralateral CA1L theta with ipsilateral CA1R gamma oscillations in seeded animals. This could result from the reduced slow gamma power at the ipsilateral CA1R around this time. There is also reduced coupling of ipsilateral CA1R theta with contralateral CA1L gamma oscillations. The impaired high-theta activity in the ipsilateral CA1R may contribute to this impairment, and the possible compensatory CA1L-enhanced theta power may not be completely effective in maintaining the normal CA1 circuit's function. This impaired intersite PAC suggests a bidirectional impairment in effective communication between both hippocampal CA1 regions.

### 5.4. Various Changes in Different Coherence Measures between Electrodes Suggest Altered Neural Network Dynamics

The main goal of coherence function is to reveal synchronous oscillations in different networks to deduce functional coupling among these networks. Highly coherent oscillations between two structures can occur because they are functionally connected or because they share a common input; e.g., coherence analysis considers all possible interactions, including those from mutual sources. However, due to a high sensitivity to noise and the possibility of volume conduction-related effects, variability can be high. K18-seeded animals showed an early deteriorating global coherence efficiency network and coherence between contralateral CA1L and ipsilateral CA1R regions for the frequency range 4-70 Hz. The shift towards slow theta observed in the power spectra may be indicatory of the beginnings of adaptive increases in intercortical connectivity and may further support the possibility of enhanced theta power to overcome impaired interhippocampal connectivity.

Partial coherence and extended PDC allowed further investigation into changes in functional network connectivity observed in the results from the standard coherence analysis. Partial coherence analysis, unlike coherence analysis, only investigates direct interactions between the two electrodes of interest and removes any possible mutual connections. Interestingly, a significant decrease in partial coherence between CA1L and CA1R was observed in tau-seeded animals at recording week 10. There are numerous physical hippocampal commissural fibers between these two regions, so this functional disconnection may not be robust enough to overcome this anatomical connectivity. Unlike with the aforementioned coherence analysis, there are no significant differences in partial coherence between FL and FR or CA3L and CA3R. This is interesting as it suggests that the changes in coherence seen between these electrode pairs may be due to changes in their coherence with mutual connections.

Extended PDC analysis is a slightly more advanced function than partial coherence and involves time lags between coherent signals, to investigate causal relationships and directionality between these network locations. Interestingly, in support of differences in partial coherence between K18- and buffer-injected animals at recording week 10 for CA1 electrode pairs, this analysis highlighted some possible directional network changes occurring. There seems to be a consistent decrease in the extended PDC index in the direction of CA1R to CA1L and other electrodes, whereas there seems to be no changes in the PDC levels in the direction of CA1L to CA1R and other electrodes. This is surprising but could be indicative of this possible compensatory activity from the contralateral CA1L region, which attempts to maintain a level of functional network connectivity with the ipsilateral hippocampus.

### 5.5. Possible Functional Biomarkers and Considerations of the Novel Brainstem Tau Pathology Mouse Model

As we have demonstrated, tau seeding in the brainstem LC was sufficient to cause early functional alterations in distant hippocampi ipsilateral to the injection site as well as the contralateral hippocampus. These functional alterations have been shown to take the form of localized changes, as well as changes in the dynamics of functional hippocampal networks. We have also seen some changes in the contralateral hippocampus to suggest a possible attempt to maintain normal function. If the contralateral hippocampus was found to be compensating for these changes, it is possible that without this compensation, the functional alterations seen in the hippocampus ipsilateral to the injection site could be far greater. As this study is focused on investigating possible functional biomarkers in this model, we have attempted to critically analyze each measure to determine its value. In this study, the earliest and most robust changes were the leftward shift to slow power spectra, impaired global coherence, and intrasite theta-gamma PAC at the CA1R. There were significant reductions seen in tau-seeded animals at 1 week postinjection, which persist throughout the recording period. Similarly, deteriorating theta-gamma PAC was found at the contralateral CA1L as well. Hippocampal theta-gamma PAC has been implicated as an important process in learning and memory in humans and rodents, giving this measure translational value [43]. This functional index may be extremely valuable, as a functional correlate of the early pathology load in live animals and how it progresses, without the need for labor-intensive immunohistochemistry. It is worth mentioning that variability in functional measures may arise from the high variability in the underlying tau pathology load between tau-seeded animals. The subsequent development of tau pathology following seeding is not completely uniform between different animals. Differences in the brain's ability to clear this pathology, as well as differences in the vulnerability of the brain to these changes, are two of many possible factors that may affect the development of tau pathology. During the stereotaxic injection of compounds into the brain, it is possible for the compound to flow back up along the needle track and out of the brain. This “backflow” could be avoided using smaller volumes of compounds, as this could result in reduced pressure at the injection site. However, one problem with reducing the amount of seed material injected into the brain could be a subsequent reduction in tau pathology.

From the translational perspective, the present model could be improved as tau pathology was unilaterally seeded in the right-side locus coeruleus, whereas tau pathology in AD initiates bilaterally, in the LC of both hemispheres [25]. To better recreate the early pathological process of AD in this model, tau pathology could be seeded bilaterally, in the LC of both hemispheres. Bilateral tau seeding in this model could also conceivably result in more severe functional changes, by removing any possible compensatory changes seen in this unilateral seeding model, and could also result in more extensive tau pathology spread within the brain. Another possible benefit of bilateral seeding could be that smaller volumes of seed material could be used, as tau spreading from both LCs may overcome the possible reduction in seeding mentioned earlier.

One final possible methodological improvement relates to the form of seed material that we have used in our model. In the previous tau-seeding models, the tau-seeding material has taken various forms. Clavaguera et al. have demonstrated the in vivo seeding potential of brain homogenates from both tau transgenic P301S mice [23] and brain extracts from humans who had died from various tauopathies [67], while we have used synthetic preformed fibrils of K18 [24]. A study by [68] found that the seeding potential of different tau aggregates is affected by the conformation of the aggregates, and as it is currently unknown which form of tau is implicated in the spread of tau between neurons, it could be beneficial to experiment with different seed materials, to determine whether this has an effect on the spatiotemporal progression of tau pathology as compared to our model. While seeding with K18 aggregates has the disadvantage of being artificial, brain homogenates may contain a few other pathogenic molecules, which could cause pathology unrelated to tau pathology. The paired helical filaments (PHFs), which are the main components of neurofibrillary tangles that are naturally found in AD, could act as a more natural seed [69].

In addition, a few other measures of health of these animals were also taken throughout the study, including body weight, 6-day food intake, and core body temperature. Regular weighing also allowed us to investigate the animal's health, as well as monitor the tau-seeded animals for the motor impairments seen in other tau transgenic mouse models with heavy hindbrain pathology. It is of note that none of the animals suffered from any hind limb clasping behavior as seen in the JNPL3 mouse model [15]. It is our view that this novel mouse model along with the robust functional alterations seen in the brains of these animals could be used as a platform for testing experimental therapeutic compounds that target tau pathology early in AD. As mentioned before, it is extremely valuable to have an indication that a therapeutic compound is resulting in a functional improvement in animals, aside from its effects on the pathology itself [11]. Moreover, with some of the possible methodological improvements mentioned earlier, this mouse model could be used to further attempt to understand the nature of the marked functional impairments that have been observed in early stages of AD using EEG rhythms. In particular, reduced EEG coherence has previously been found in individuals homozygous for the APOE *ε*4 allele [70]. EEG/EMG connectivity data have been shown to correlate with structural and functional hallmarks of neurodegeneration such as hippocampal atrophy [71]. In recent years, many studies have combined EEG/MEG techniques to graph theories to investigate brain connectivity in AD [9, 41, 72–78]. AD patients are characterized by loss of EEG synchrony among distributed functional networks [79] and significantly reduced interhemispheric theta coherence [79]. Other studies reported the increased delta coherence, decreased theta and alpha coherence, higher alpha and lower delta, and beta small world characteristics of connectivity [80, 81]. A global reduction of functional long-distance brain connectivity and transhemispheric coherence were found in EEG resting states of AD patients [82]. A decreased coherence of high-frequency gamma rhythms has been identified as a specifically predictive sign of conversion from MCI to initial AD [83]. In addition, reduced complex EEG activity and decrease in coherence of fast EEG rhythms were described in EEG of AD patients [9, 84, 85]. Further evidence for a close relation between dysfunction of cortical connectivity and AD comes from studies investigating effective connectivity of neuronal systems based on Granger causality estimates. Reduced effective connectivity in high-frequency rhythms from parietal to frontal electrodes was observed in AD and MCI patients [86, 87].

Additional newly emerging stimulation techniques such as noninvasive transcranial magnetic stimulation (TMS) have proven reliability in probing selective impairment of specific intracortical circuits in neurodegenerative disorders with primary or secondary tauopathy and in assessing the underlying neurotransmitter deficits [88]. Cerebrospinal fluid tau levels mediate abnormal cortical excitatory activity [89], associated with prominent long-term depression (LTD) mechanisms of cortical plasticity and faster cognitive decline [90]. In animal models of AD, hippocampal long-term potentiation (LTP), which is an electrophysiological correlate of learning and memory, is impaired by A*β* peptides and tau proteins [91–93]. New models of AD pathophysiology have suggested a primary dysfunction of midbrain catecholaminergic systems [94–96]. A*β*-induced dopamine depletion has been suggested as a core mechanism underlying the early synaptopathy and memory alterations observed in AD models and acts by modifying the threshold for the induction of cortical LTP and/or LTD [97]. Dopamine agonists could restore the altered mechanisms of LTP-like cortical plasticity in AD patients, thus providing novel implications for therapies based on dopaminergic stimulation [98]. Early loss of noradrenergic drive contributes to the impairment of cerebellar synaptic plasticity and motor learning [99, 100], and restoration of the noradrenergic tone has been shown to reverse these effects and slow neurodegeneration in animal models of AD [101, 102]. Cortical plasticity measures such as LTP/LTD can be obtained by applying noninvasive repetitive TMS over the primary motor cortex, using theta burst stimulation protocols that mimic those described in animal models. TMS combined with simultaneous EEG provides unique possibilities to study and map the excitability and plasticity of the brain as well as its functional connectivity in a much deeper and time-resolved manner [103–107]. The primary disintegration of hippocampal networks observed in the present mouse model of tauopathy could be used in translational studies in samples of populations with primary tauopathies or of AD patients with high tau accumulation in the brain. Simultaneous recording of TMS-EEG thus offers an important means of directly testing the model.

## 6. Conclusion

To conclude, we have seen numerous robust functional changes in the hippocampus of mice seeded with K18 aggregates in the deeper brainstem LC area. These changes are indicative of decreasing neuronal activity at the ipsilateral hippocampus, along with some possible compensatory increases in neuronal activity in the contralateral hippocampus, alterations in the functional connectivity of specific functional neural network connections, and decreased theta-gamma PAC, suggesting impaired synaptic plasticity. It is our hope that these results will highlight to the field the relevance of the development of tau pathology in the brainstem early in AD and challenge the belief that it is the spread of tau pathology to a region of the brain in tauopathy which is the necessary sole causal factor for severe functional deterioration of this region.

## Data Availability

All relevant data within the article are fully available.
